# Practical tips for managing myopia

**Published:** 2019-05-13

**Authors:** Michael Morton, Ling Lee, Priya Morjaria

**Affiliations:** 1 Online Education Coordinator: Brien Holden Vision Institute, Sydney, Australia.; 2 Research Officer/Optometrist: Brien Holden Vision Institute, Sydney, Australia.; 3 Research Fellow: Department of Clinical Research, London School of Hygiene and Tropical Medicine, International Centre for Eye Health, London, UK.

**This article presents a summary of practical approaches to diagnosing myopia, myopia management (with particular attention to low resource settings), reviewing myopia progression, and collecting data for myopia management programmes.**

**Figure:**
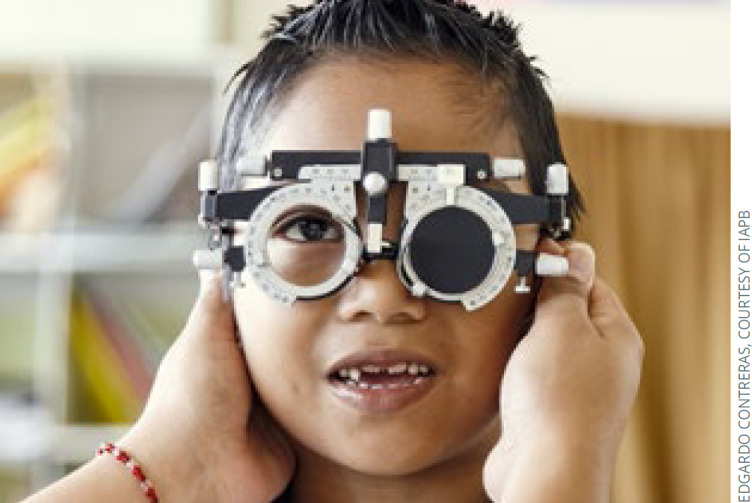
Refraction is the first step. MEXICO

## Part 1 Diagnosing and prescribing for myopia

While myopia might be initially detected by a patient (e.g. reporting distance blur), or an adult observing behaviour changes in a child (e.g. squinting or viewing things closer than expected), myopia is generally diagnosed by an eye care professional.

### Equipment

The minimum required equipment to diagnose myopia and assess progression includes:

- A high-contrast distance visual acuity (VA) chart (e.g., Snellen, logMAR, E, or LEA)
- A room or space where the viewing distance for VA is at least 3m/10ft. The chart should be well lit and calibrated for the working distance
- Occluder (ideally with pinhole occluder)
- Retinoscope
- Trial lens set (including Jackson Cross Cylinder) or phoropter

Equipment that could assist with myopia diagnosis, management and estimation of progression:

- Near VA chart
- Autorefractor
- Heterophoria measurement method (e.g., MIM card or Howell phoria card)
- Lens flippers (±1.00 and ±2.00 D)
- Prism bars
- Optical biometer for axial length measurement
- Cycloplegic eye drops (e.g., tropicamide, cyclopentolate or atropine).

### Clinical techniques

To prescribe for myopia appropriately, the clinical techniques below are recommended as a minimum:

- Visual acuity (VA)
- Retinoscopy
- Subjective refraction
- Ocular health assessment.

The following clinical techniques are recommended and should be conducted where possible:

- Cycloplegic refraction / autorefraction
- Axial length measurement.

The binocular vision tests below can measure the effect of a myopia management strategy on the individual's binocular vision status and visual comfort. This will help you to determine the appropriateness of a myopia management strategy for that person.

- **Monocular estimate method (MEM) retinoscopy**. An objective method to determine a child's accommodative (near focussing) status at near. Retinoscopy should be conducted with a near target.
- **Accommodative facility.** A subjective method to assess accommodation function (ability of eye to focus at near).
- **Subjective phorias.** A subjective method to determine whether the eyes prefer to converge in or diverge out, at distance and near.
- **Vergence reserves.** A subjective method that measures the eyes' ability to converge in and diverge out.
- **AC/A ratio.** Assesses the interaction between accommodation and convergence.

### Cycloplegic refraction

An accurate refraction is very important in diagnosing myopia and monitoring myopia progression. Ideally, a child with myopia should undergo cycloplegic refraction at the initial presentation and then at least every 12 months. This allows the clinician to accurately determine the refractive error without the effect of an active accommodation system. However, if cycloplegic refraction is not possible, a careful subjective refraction must suffice.

Common agents used for cycloplegia include cyclopentolate (0.5% or 1%), tropicamide (0.5% or 1.0%) and atropine (1%). Studies have found tropicamide 1% to be effective in monitoring standard cases of myopia progression.^1^,^2^ Therefore, tropicamide 1% is worth considering for cycloplegic refraction as it reduces the duration of glare and near symptoms compared to other options.

If conducting cycloplegic refraction, inform patients that the eye drops may sting for a few seconds before instilling them into the eye. Advise that their vision may be blurred, especially at near, and they may be light sensitive for a few hours, so sunglasses should be worn if possible.

### What should you prescribe?

Use the cycloplegic refraction results from retinoscopy or autorefraction as the starting point and then refine to achieve the best possible VA. If there is a large difference between the cycloplegia objective and subjective refraction, recheck your results.

#### Uncorrected astigmatism

In low-resource settings where sphero-cylindrical lenses are unavailable to correct astigmatism, spherical lenses might be prescribed to correct myopia. The level of blur is dependent on the amount and type of astigmatism and currently there is no evidence on the effect of uncorrected astigmatism on myopia progression.

#### Under-correction is ineffective

Studies show prescribing single vision lenses with under-correction (less minus) made no difference compared to full correction, and in some cases, it made the myopia progression worse.^3^,^4^ This suggests that if you are only able to prescribe single vision spectacles, full correction is recommended.

## Part 2 Options for managing myopia

For children at risk of developing myopia, advise at least 90 minutes of outdoor time daily,^5^ and regular breaks from near work.

Myopia management interventions for children with progressive myopia can be divided into three categories (Table 1). These may not all be available in low- or middle-income countries. Each intervention is generally prescribed alone. However, you could consider combination treatment (for example, adding low-dose atropine to an optical treatment) if there is a high risk of fast progression or poor response to individual treatment. You can prescribe single vision lenses if you consider the patient's myopia to be stable, especially if the patient is an adult.

**Table 1.** Myopia management interventions

Pharmacological	Optical: spectacles	Optical: contact lenses
Low-dose atropine (might require a specialist compounding pharmacy)	Executive bifocals Progressive addition lenses	Dual-focus and multifocal centre-distance contact lenses Orthokeratology

### Choosing a myopia management strategy

Consider:

- The options are available in your setting
- Their effectiveness
- Patient suitability
- Patient and carer preference

Using your clinical judgement, decide which of the available options is most appropriate for your patient. If you are unable to prescribe that option, refer to a colleague who has experience with, and access to, that intervention.

### What next?

#### Review periods

Review every 6 months. More frequent reviews may be required for patients when first introduced to each treatment strategy. Ongoing follow-up should be based on the patient's progress, treatment modality and performance.

#### What is fast progression?

Fast progression is progression of myopia of 1.00 D or more, per year.^6^ Younger children with myopia are more likely to progress faster than older children.^7^

### How to estimate progression

You can estimate progression by comparing the difference in myopia before and after using a myopia management strategy. Ideally, you will be comparing cycloplegic refractions over at least 12 months. Table 2 is an example of estimating the rate and reduction of myopia progression:

**Table 2.** Recording and estimating the rate of progression: an example

Examination date	Age	Refraction
3 years ago	7	R −1.00 D L −0.75 D
2 years ago (started myopia management)	8	R −2.00 D L −1.75 D
Today	10	R −3.00 D L −3.00 D

- Prior to using myopia management, this patient had progressed −1.00 D in one year.
- With myopia management, they then progressed to R (right eye) −1.00 D and L (left eye) −1.25 D over 2 years, which is −0.50 D and −0.62 D per year, respectively.
- This in an approximate reduction of 50% in the right eye and 38% in the left eye.

Estimating progression might be difficult for patients prescribed orthokeratology, and measuring axial length might assist in these cases. However, you need to be aware that axial length also increases with age in children with emmetropia (normal eye length).^8^

### Data collection for myopia management

Record keeping is important to monitor a child's myopia onset, progression, and their response to treatment. This information can also assist in developing a database to use when planning programmes. Record:

- The child's age at the onset of myopia (-0.50 D in either eye) from a cycloplegic refraction
- Ethnicity
- Family history of myopia
- Number of hours or minutes spent outdoors each day
- Number of hours or minutes spent on near work
- The type(s) of myopia management prescribed
- When the myopia management was initiated/ceased
- The amount of myopia (i.e. prescription) at each visit, at least annually, with cycloplegia
- Axial length (where possible).

### Summary: managing myopia in low- or middle-income countries

- It is important that progressive myopia is diagnosed and managed appropriately.
- Know which myopia management strategies are available in your setting (such as executive or large segment bifocal spectacles, multifocal contact lenses, orthokeratology and low-dose atropine).
- Consider which myopia management strategy would be most appropriate for your patient.
- If that option is within your scope of practice, prescribe it. If not, refer your patient to a colleague who can prescribe it.
- Compare the annual rate of progression with and without myopia management using subjective refraction, ideally with cycloplegia.
